# The role of extracellular matrix components in pin bone attachments during storage—a comparison between farmed Atlantic salmon (*Salmo salar*) and cod (*Gadus morhua* L.)

**DOI:** 10.1007/s10695-016-0309-0

**Published:** 2016-11-02

**Authors:** Sissel B. Rønning, Tone-Kari Østbye, Aleksei Krasnov, Tram T. Vuong, Eva Veiseth-Kent, Svein O. Kolset, Mona E. Pedersen

**Affiliations:** 1 Nofima AS, Pb 210, 1431 Ås, Norway; 2 Department of Nutrition, Institute of Basic Medical Sciences, University of Oslo, Oslo, Norway

**Keywords:** Pin bone, Extracellular matrix, Proteoglycans, Connective tissue

## Abstract

Pin bones represent a major problem for processing and quality of fish products. Development of methods of removal requires better knowledge of the pin bones’ attachment to the muscle and structures involved in the breakdown during loosening. In this study, pin bones from cod and salmon were dissected from fish fillets after slaughter or storage on ice for 5 days, and thereafter analysed with molecular methods, which revealed major differences between these species before and after storage. The connective tissue (CT) attaches the pin bone to the muscle in cod, while the pin bones in salmon are embedded in adipose tissue. Collagens, elastin, lectin-binding proteins and glycosaminoglycans (GAGs) are all components of the attachment site, and this differ between salmon and cod, resulting in a CT in cod that is more resistant to enzymatic degradation compared to the CT in salmon. Structural differences are reflected in the composition of transcriptome. Microarray analysis comparing the attachment sites of the pin bones with a reference muscle sample showed limited differences in salmon. In cod, on the other hand, the variances were substantial, and the gene expression profiles suggested difference in myofibre structure, metabolism and cell processes between the pin bone attachment site and the reference muscle. Degradation of the connective tissue occurs closest to the pin bones and not in the neighbouring tissue, which was shown using light microscopy. This study shows that the attachment of the pin bones in cod and salmon is different; therefore, the development of methods for removal should be tailored to each individual species.

**Electronic supplementary material:**

The online version of this article (doi:10.1007/s10695-016-0309-0) contains supplementary material, which is available to authorized users.

## Introduction

False ribs, also called pin bones, are bones that extend into the muscle tissue. So far, little is known about how the pin bones are attached to the muscle and if there are differences in biological composition and morphology between salmon and whitefish. The connective tissue (CT) of fish is composed of cells and extracellular matrix (ECM), in addition to blood vessels and nerves. The CT helps to attach the pin bones to the muscle, and the strength of the CT is determined by the composition and organisation of the different ECM components (Carmeli et al. 2004). The CT is a highly dynamic structure and may change over time in conjunction with increased/decreased stress, altered nutrient intake, age etc. (Tingbo et al. 2012a; Danielson et al. 1997). Normal physiological processes in fish are dependent on the precise remodelling of the ECM, which is composed of proteoglycans (PGs) and fibrous proteins, with collagen being the most abundant protein. The ECM provides mechanical support, and it signals to the interior of the cell, affecting a variety of cellular responses. The ECM is constantly undergoing changes in response to cellular stimuli, with a well-adjusted interplay between synthesis and deposition of ECM components on one hand and their proteolytic breakdown on the other. Degradation of the CT is enzymatic, and enzymes involved are affected by for example ion concentrations and pH (Vargova et al. 2012; Nguyen et al. 1990). Some ECM components degrade more readily than others.

The unwanted bones are a major challenge for aquaculture (salmon) and fishing (whitefish) industry. At present, removal is expensive and difficult; the main problems are damage of the fillet and fracture of the bones inside the fillet. There are also major differences between the fish species in terms of bone strength and pulling force required to remove the pin bones (Esaiassen and Sørensen 1996; Akse and Tobiassen 2002; Westavik 2009). The precise identification of the CT components is important in order to characterise the physiology of pin bones, information that possibly could help the industry to develop methods for efficient pin bone removal. To achieve this, it is necessary to identify how the pin bones are attached, the attachment structures and the degradation of these.

## Materials and methods

### Antibodies

Sheep anti-Decorin (ab35378-1), mouse anti-Lumican (ab70191) and rabbit anti-Collagen I were from Abcam (Cambridge, UK). Mouse anti-C-4-S (2B6) and mouse anti-C-6-S (3B3) were from Millipore (Billerica, MA, USA). Mouse anti-C-0-S (1B5) was from Northstar BioProducts (MA, USA—formerly Seikagaku America). Alexa Fluor 488-conjugated goat anti-rabbit, Alexa Fluor 546 conjugated goat anti-mouse and Alexa Fluor 488-conjugated donkey anti-sheep were from Invitrogen (Carlsbad, CA, USA). DAPI and Alexa Fluor 594-conjugated wheat germ agglutinin (WGA) were from Molecular Probes (Invitrogen, Carlsbad, CA, USA).

### Sampling

Farmed Atlantic salmon (*Salmo salar* L.) originating from the breeding company SalmoBreed AS, Norway and Atlantic cod (*Gadus morhua* L.) with parents of first generation offspring from wild-caught stem fish were used. The farmed salmon (3.5 kg) and cod (4 kg) were treated as production fish up to sacrifice at Nofima research station (Averøy, Norway) and Havbrukstasjonen (Tromsø, Norway) respectively. The fish were anesthetised with MS222 (Norsk Medisinaldepot, Oslo, Norway) and then killed by cutting of the gills. Fillets harvested immediately after slaughter were stored on ice for either 60 min or 5 days, the pin bones were dissected and then fixed or frozen in liquid nitrogen. Samples for microarray were as follows: pooled samples were made from the two foremost and the two hindmost pin bones from fillets of Atlantic salmon (*n* = 8) and Atlantic cod (*n* = 4). For the microarray study, the pin bones were excised immediately after slaughter and muscle samples from the same region were used as reference. For proteome analysis (*n* = 6), pin bones were excised from the foremost regions of the fish fillets, frozen in liquid nitrogen and stored at −80 °C until further analysis. For the microscopy study (*n* = 4), pieces including pin bone area of approximately 15 × 10 × 10 mm were cut from the same area as samples for microarray in fish fillets and fixed in ZBF containing 36.7 mM ZnCl_2_, 27.3 mM ZnAc_2_ × 2H_2_O, 0.63 mM CaAc_2_ in 0.1 M Tris and pH 7.4 for 36–38 h. Thereafter, the samples were decalcified with EDTA (14 %, pH 7.1) for 10 days, before dehydration and paraffin embedding. The 5-day storage samples were collected from different individuals than the 60-min samples but at the same morphological location.

### Histology

Five-micrometre-thick sections of fixed, paraffin-embedded samples were cut on a paraffin microtome (Leica RM 2165, Germany) and mounted on poly-l-lysine-coated glass slides. Histological analyses were carried out on deparaffinised sections: 2 × 5 min in xylene before rehydration in series of ethanol before rinsing with dH_2_O. To outline the structure of the pin bone and its surrounding connective tissue, toluidine blue (1 % toluidine blue/70 % alcohol diluted 10× in 1 % sodium chloride) was used as a staining protocol. The sections were immersed in staining solution at room temperature for 3 min, rinsed in running water, dehydrated in absolute ethanol and mounted in Eukitt. To monitor the presence of sulphated glycosaminoglycans, Alcian Blue 8GX (Gurr Biological Stains, BDH, Poole, UK), 0.05 % in 0.2 M Na acetate buffer, pH 5.8, with 0.4 M MgCl2, was used as a staining solution. The sections were immersed in staining solution at room temperature with gentle shaking overnight, rinsed in running water, dehydrated in absolute ethanol and mounted in Eukitt. Verhoeff-van Gieson staining protocol was used for staining of elastic tissue fibres. Sections were stained for 30 min with Verhoeff’s haematoxylin, rinsed in dH_2_O and differentiated in 2 % ferric chloride for 2 min to remove haematoxylin in other compartments than elastic tissues. Sections were rinsed in running water, dehydrated, cleared and mounted in Eukitt.

### Immunohistochemistry

Sections were dehydrated in decreasing ethanol concentrations before permeabilisation with 0.5 % Triton X-100 in 1× PBS for 15 min, before blocking in 5 % non-fat dry milk powder dissolved in 1× PBS. The primary antibodies diluted in 2 % non-fat dry milk in PBS were incubated overnight at 4 °C before washing with 1× PBS for 30 min (Collagen I 1:40, Decorin 1:100, Laminin 1:10 and Lumican 1:100). Subsequent incubation with secondary antibodies was performed for 2 h, washing with 1× PBS for 30 min before using Dako fluorescent mounting medium (Glostrup, Denmark). The sections were co-stained with Alexa Fluor 488 WGA (a probe that labels sialic and *N*-acetylglucosaminyl residues). The cells were examined by fluorescence microscopy analysis (ApoTome mode) (Zeiss AxioObserver Z1 microscope, Jena, Germany), and images were processed using Adobe Photoshop CS3. Brightness and contrast, if used, were adjusted manually across the entire image. The objective used with fluorescence microscopy was a LCI Plan-Neofluor 25×/0.8 1 mm Korr M277 objective oil.

For identification of the different sulphated structures present in the connective tissues, the following antibodies were used: mAb 2B6 for detection of C-4-S, mAB 1B5 for detection of C-0-S and mAB 3B3 for C-6-S, all diluted 1:100 in 2 % non-fat milk. To generate the anti-genic epitopes, the sections were digested with chondroitinase ABC lyase (cABC) from *Proteus vulgaris* (0.5 units/mL) in 0.1 M Tris-HCl buffer, pH 8. After cABC treatment for 2 h at 37 °C, non-specific binding was blocked by using 5 % non-fat dry milk powder dissolved in 1× PBS. IHC was performed as described above.

### Microarray analysis

RNA was extracted using PureLink RNA Mini kits according to the manufacturer’s protocol (Invitrogen, CA, USA). Concentration of total RNA (NanoDrop 1000 Spectrometer, Thermo Scientific, Waltham, MA, USA) and RNA integrity were measured (Agilent 2100 Bioanalyzer with RNA Nano kits, Agilent Technologies, Santa Clara, CA, USA). Samples with RNA integrity number (RIN) >8 were accepted for analyses. Multiple gene expression profiling was performed with the following oligonucleotide microarrays: Atlantic salmon 15 k SIQ6 (GEO Omnibus GPL16555) and genome-wide Atlantic cod 44 k ACIQ1 (GEO Omnibus GPL18779). The microarrays were designed by Nofima (Krasnov et al. 2011, 2013) and produced by Agilent Technologies. Individual pin bone samples were labelled with Cy5 and hybridised to pooled muscle sample labelled with Cy3; a total of 16 microarrays were used. RNA amplification, labelling and fragmentation were performed using the Two-Colour Low Input Quick Amp Labelling Kit and Gene Expression Hybridization Kit following the manufacturer’s instructions (Agilent Technologies). The input of total RNA in each reaction was 100 ng. Overnight hybridisation (17 h, 65 °C and a rotation speed of 10 rpm) was executed in an oven (Agilent Technologies). The slides were washed with Gene Expression Wash Buffers 1 and 2 and scanned with a GenePix 4100A (Molecular Devices, Sunnyvale, CA, USA) at 5-μm resolution. The GenePix Pro software (version 6.1) was used for spot to grid alignment, feature extraction and quantification. Assessment of spot quality was done with GenePix flags. Nofima’s bioinformatics package STARS (Krasnov et al. 2011) was used for data processing and mining. Differentially expressed genes (DEG) were selected as log2-ER > |1| (twofold) and *p* < 0.01 (one-sample *t* test). All the presented microarray data are significant as explained in the text.

### Proteome analysis

The connective tissue surrounding 1–2 pin bones (approximately 100 mg) from a total of six fish per sampling time were extracted using a three-step protocol, starting with a Tris buffer (10 mM Tris, pH 7.6, 1 mM EDTA, 0.25 M sucrose), followed by NaCl buffer (0.5 M NaCl, 10 mM Tris, pH 7.6) and finally a urea buffer (7 M urea, 2 M thiourea, 2 % CHAPS, 1 % DTT). First, the frozen tissue was homogenised in 1 mL Tris buffer using a Precellys 24 (Bertin Technologies, Villeurbanne, France) at 5500 rpm for 2 × 20 s, followed by centrifugation (30 min at 7800 g, Heraeus, Biofuge Fresco, Hanau, Germany) at 4 °C and discarding of the supernatant. The remaining pellet was rehomogenised in 1 mL Tris buffer using the same conditions as above. After having repeated this step twice, the pellet was rehomogenised in the NaCl buffer with three repeats, and finally, the pellet was rehomogenised in the urea buffer. This homogenate was then shaken vigorously for 1 h at room temperature followed by a final centrifugation to remove any insoluble components. Protein concentrations were measured with a commercial kit at 750 nm (RC DC Protein Assay, Bio-Rad) in a spectrophotometer with BSA as standard.

Isoelectric focusing was performed using immobilised pH gradients (pH 5–8, 24 cm) and the Ettan IPGphor II unit (GE Healthcare Bio-Sciences, Uppsala, Sweden). Initially, a low voltage (100 V) was applied, followed by a stepwise increase to 8000 V, reaching a total of ∼80,000 Vh. In the second dimension, proteins were separated on 12.5 % SDS-PAGE using the Ettan DALTtwelve large format vertical system (GE Healthcare Bio-Sciences). For analytical gels, 100-μg protein was loaded for each sample, and the protein spots were visualised by Blum’s silver staining (Blum et al. 1987), while the preparative gels were loaded with 500-μg protein and visualised using the Shevchenko silver staining protocol (Shevchenko et al. 1996). Image analysis was performed using Progenesis SameSpots version 4.5 (Nonlinear Dynamics Ltd., Newcastle upon Tyne, UK), and the statistical tools within this software were used to reveal significantly altered protein spots between the two sampling time points: i.e. regular ANOVA, resulting in *p* values, and adjusted *p* values calculated using a false discovery rate approach, resulting in the more stringent *q* values.

Significantly altered protein spots were excised from preparative 2-DE gels for trypsin treatment and peptide extraction, and the resulting peptide mixtures were desalted and concentrated using small discs of C18 Empore Discs (3M, USA) (Gobom et al. 1999). Peptides were eluted with 0.8 μl matrix solution (α-cyano-4-hydroxycinnamic acid (Bruker Daltonics, Germany) saturated in a 1:1 solution of ACN and 0.1 % TFA) and spotted directly onto a matrix-assisted laser desorption/ionisation time-of-flight (MALDI-TOF) target plate. An Ultraflex MALDI-TOF/TOF mass spectrometre with a LIFT module (Bruker Daltonics) was used for mass analyses of the peptide mixtures. FlexAnalysis (version 3.4, Bruker Daltonics) was used to create the peak lists, and BioTools (version 3.2, Bruker Daltonics) was used for interpretation of MS and MS/MS spectra. Proteins were identified by peptide mass fingerprinting using the database search programme Mascot (http://www.matrixscience.com), and the following search parameters were used: MS tolerance of 50 ppm, MS/MS tolerance of 0.5 Da, maximum of missed cleavage sites was one and carbamidomethyl (*C*) and oxidation (*M*) were used as fixed and variable modifications respectively.

## Results

### Differences in gene expression in the pin bone area compared to the muscle of cod and salmon

Difference between the pin bone areas (pin bone, CT and surrounding muscle) compared to surrounding reference muscle sample was much greater in cod than in salmon as seen by the number of DEGs: 1885 and 185 features respectively (Table 1). In both species, differences between the anterior and posterior pin bones were minor: the number of DEG were 7 (3.8 %) in salmon and 61 (3.8 %) in cod. Difference between the species was also seen when comparing functional groups among DEG (Table 2). Compared to the surrounding reference muscle tissue, the pin bone area showed significant changes in the structure of the striated muscle in cod: 56 and 84 muscle-specific genes were upregulated and downregulated respectively, when compared with surrounding reference muscle tissue. The greatest changes were shown for alpha-tropomyosin 3 (tpm3, 110-fold higher expression) and cardiac muscle chain 6 alpha (myhz, 21.7-fold lower expression, Table S1). Members of several multigene families showed an opposed trend to each other: the most striking of which were two isoforms of same gene; troponin I (tnni), which were 32.2-fold overexpressed and 11.9-fold underexpressed (Table S1). In salmon, expression changes in the pin bone areas were shown for only four myofibre proteins and a muscle-specific calcium transporter *atp2a1*. In both species, the pin bone areas were characterised by higher expression of collagens and several other proteins of extracellular matrix. In salmon, the greatest difference (45-fold) was shown for type X collagen, which is produced by chondrocytes during ossification. Cod pin bone area showed higher expression of transporters involved in bone formation (slc16a4 and slc4a5). A number of regulators of differentiation were activated in both species, while rnasel3, which plays a key part in angiogenesis, was one of the most downregulated genes in salmon in the pin bones areas. A noteworthy difference between the species was a strong decrease of multiple secretory proteins in salmon, while several plasma proteins were upregulated in cod.

**Table 1:** Summary of genes with expression differences in cod and salmon

	Salmon	Cod
Differentially expressed genes (DEGs)	185	1887
Higher expression in pin bone area	102	863
Difference between anterior and posterior regions	7	61

Atlantic salmon (*n* = 8) and Atlantic cod (*n* = 4) samples from pin bone areas (pin bone, CT and surrounding muscle) were compared to surrounding reference muscle

**Table 2:** Presentation of functional groups in DEG genes were annotated in STARS (Krasnov et al. 2011)

Category	Salmon		Cod	
	Up	Down	Up	Down
Chromosome maintenance and modification	0	0	2	8
DNA metabolism	0	0	0	7
Protein folding and modification	0	3	9	0
Myofibre	1	2	56	84
Response to oxidative stress	0	1	0	10
Stress response	0	5	2	0
Transcription, RNA processing	0	0	5	27
Cell transport	0	0	5	6
Acute phase response	0	8	2	2
Metabolism of calcium	0	0	5	6
Metabolism of ions	0	0	5	8
Metabolism of lipids	6	0	12	5
Mitochondria	0	0	42	20
Metabolism of nucleotides	0	0	1	8
Proteases	8	2	2	5
Protein biosynthesis	0	0	3	48
Metabolism of steroids	4	0	3	6
Metabolism of sugars	0	0	4	9
Secretory proteins	0	12	6	1

Atlantic salmon (*n* = 8) and Atlantic cod (*n* = 8) samples from pin bone areas (pin bone, CT and surrounding muscle) were compared to surrounding reference muscle

There was no sign of inflammation in the pin bone area, and the amount of differentially expressed immune genes was small in both species (Tables 2 and S1). While the number of upregulated and downregulated genes was similar in cod, several acute phase proteins showed sharp decline in salmon. The pin bone areas of cod showed greater expression of several heat shock proteins and Jun transcription factors, master regulators of cellular stress in bony fish, while a panel of genes involved in responses to oxidative stress were downregulated. Several stress-related genes including four Jun paralogs were differentially expressed in salmon, and all were downregulated. In cod, genes for enzymes and proteins of lipid metabolism changed expression in both directions, while genes of steroid metabolism were reduced (Table S1). Apart from apolipoproteins that were downregulated in concert with other secretory proteins, a tendency to stimulation of genes involved in lipid and steroid metabolism was evident in salmon pin bone areas. In parallel, several genes involved in biotransformation of endogenous and exogenous lipophilic substances were upregulated. Multiple genes for cellular structures and processes were affected only in cod (Tables 2 and S1). Of note is the downregulation of genes involved in DNA replication and maintenance of chromosomes, transcription and processing of RNA. A higher number of genes for mitochondrial proteins were upregulated. In contrast, massive decrease of expression was seen in genes involved in nucleotide metabolism and protein biosynthesis.

### Pin bones are connected to the surrounding tissue with both strong and weak extracellular matrix components

**Fig. 1:**
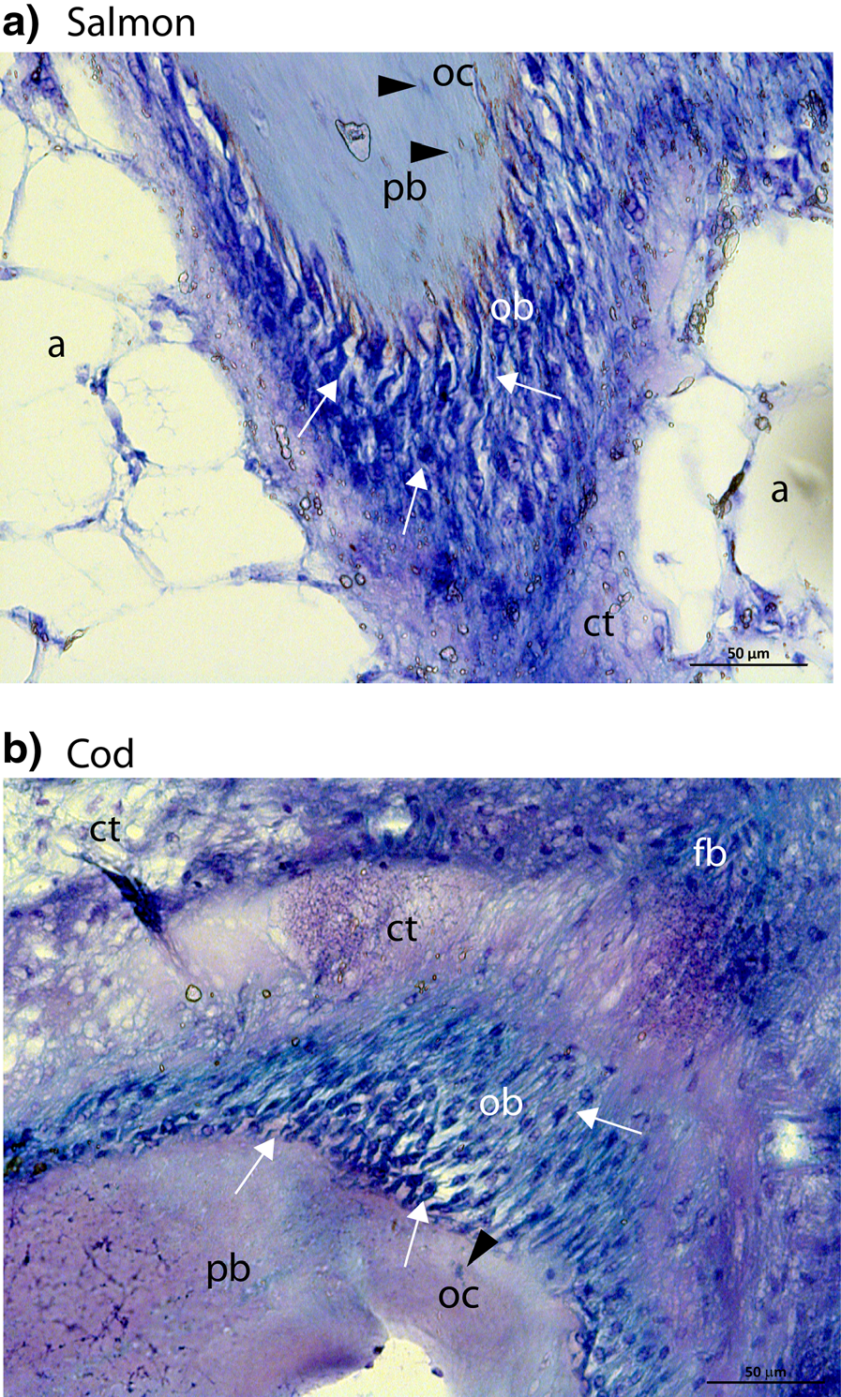
Morphological analysis of the growth zone of the tip of the pin bone. **a**, **b** Toluidine blue staining of the growth zone of pin bone in salmon (*upper panel*, **a**) and cod (*lower panel*, **b**). A dense layer of osteoblasts (bone producing cells) surrounding the pin bone is observed, indicated by *arrows*. Osteocytes are osteoblasts incorporated in the pin bone, indicated by *arrowhead*. *pb* pin bone, *a* adipose tissue, *ct* connective tissue, *ob* osteoblasts, *oc* osteocyte, *fb* fibroblast. *Scale bars* as indicated

**Fig. 2:**
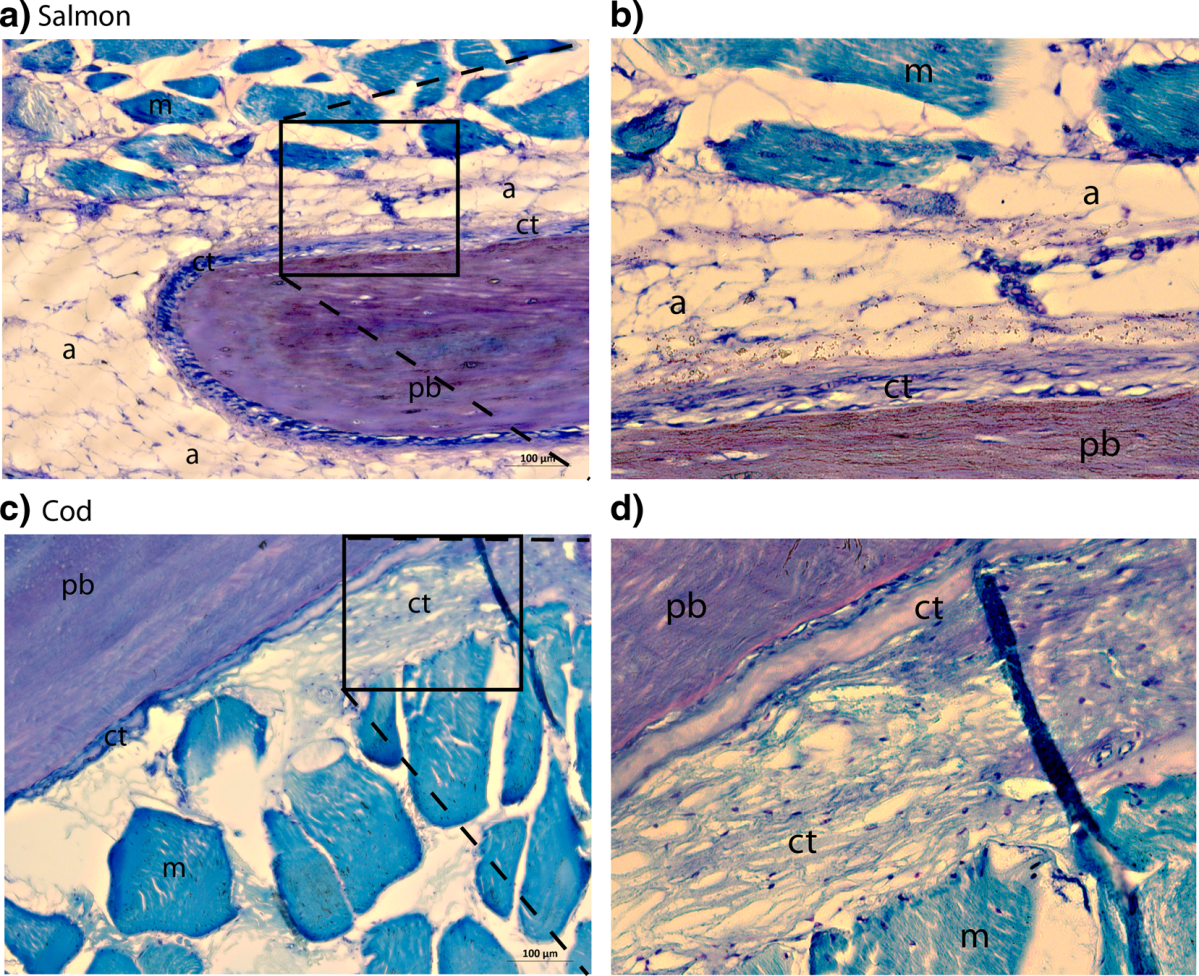
Morphological analysis of the attachment areas of pin bones in salmon and cod. **a**–**d** Toluidine blue staining of the pin bone attachment in salmon and cod. **a** The pin bone in salmon is tightly attached to adipose tissue via the CT which in turn is attached to the muscle tissue. **b** Higher magnification of *boxed area* in **a**. **c** Staining as **a** in cod. The pin bone in cod is firmly connected to the muscle tissue via CT. Note that no adipose tissue is present between the CT and the muscle tissue. **d** Higher magnification of *boxed area* in **e**. *pb* pin bone, *a* adipose tissue, *ct* connective tissue, *m* muscle tissue. *Scale bars* as indicated

**Fig. 3:**
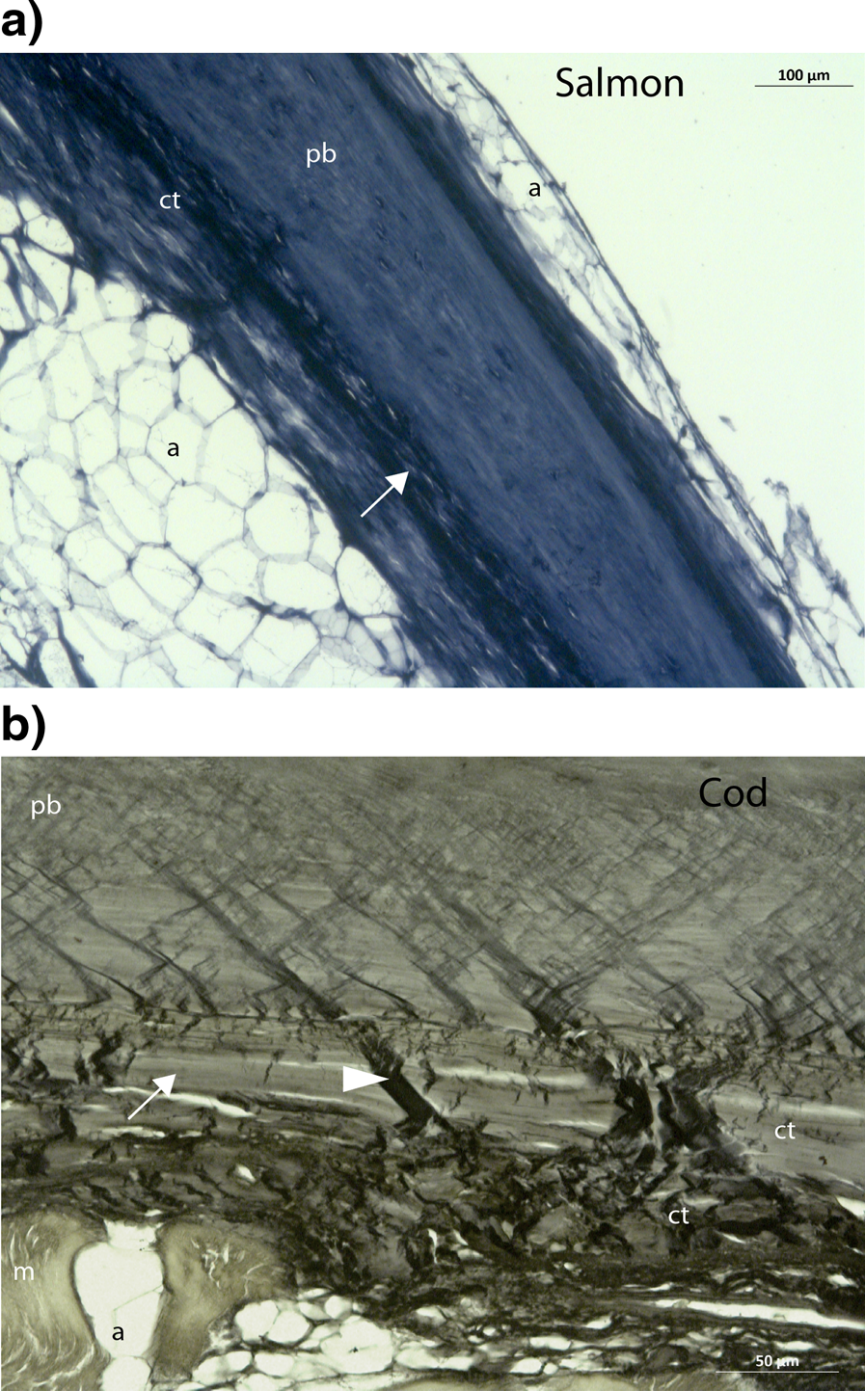
The attachment areas of pin bones in salmon and cod are rich on elastin. **a**, **b** Verhoeff’s haematoxylin staining of the elastic membrane in salmon (**a**) and cod (**b**). The pin bone and the connective tissue are rich in elastin. An elastic membrane surrounds completely the pin bone (highlighted with *arrows*). Also, elastin structures can be observed crossing the elastic membrane that surrounds the pin bone (*arrowheads*). *pb* pin bone, *a* adipose tissue, *ct* connective tissue, *m* muscle tissue. *Scale bars* as indicated

**Fig. 4:**
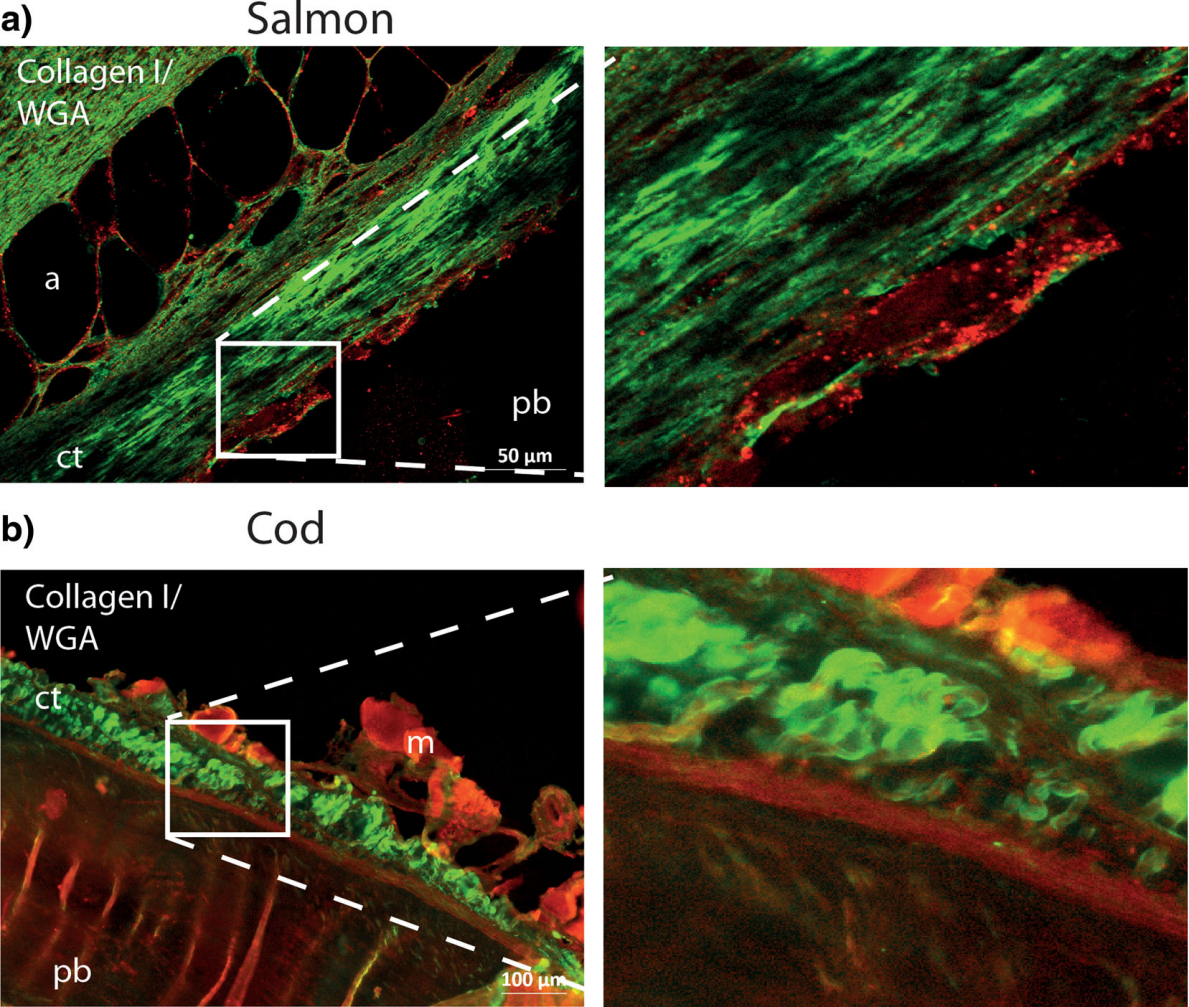
Collagen I and carbohydrate-binding proteins are present in the attachment areas in salmon (**a**) and cod (**b**). **a**, **b** Zn-fixed longitude sections of pin bone attachment sites were stained with rabbit anti-collagen 1 (*green*) and Alexa Fluor 594 WGA (*red*; binds to sialic acid and *N*-acetylglucosaminyl residues) followed by Alexa Fluor 488-conjugated goat anti-rabbit before fluorescence microscopy analyses. The *boxed area* presented at high magnification at the *right upper and lower panels* demonstrates collagen I staining and a dense area of carbohydrate-binding proteins (WGA) that surrounds the pin bone. *Scale bars* as indicated *pb* pin bone; *a* adipose tissue; *ct* connective tissue; *m* muscle tissue

Morphological analyses of the pin bone in salmon (Fig. 1a) and cod (Fig. 1b) showed an active growth zone at the tip of the pin bone, consisting of a dense layer of bone producing cells (osteoblasts) surrounding the pin bone. Osteocytes within the pin bone were also observed. The extracellular matrix of the bone is synthesised and secreted by these osteoblasts. The attachment site of the pin bones in salmon contained CT and a layer of adipose tissue before the muscle tissue (Fig. 2a, b). In cod, on the other hand, the pin bone was firmly attached directly to the muscle tissue via the CT (Fig. 2c, d). To further characterise the components in the CT, we stained for various matrix proteins, and our analyses demonstrated the presence of elastin in the CT around the pin bone in both salmon and cod (Fig. 3a, b). Collagen is the most abundant fibrous protein in the ECM, and immunohistochemical analyses showed that collagen I was present in the CT around the pin bone (Fig. 4a, b). Interestingly, when co-staining for sialic acid and *N*-acetylglucosaminyl residues using WGA was performed, we observed a strong staining in the CT area closest to the pin bone.

**Fig. 5:**
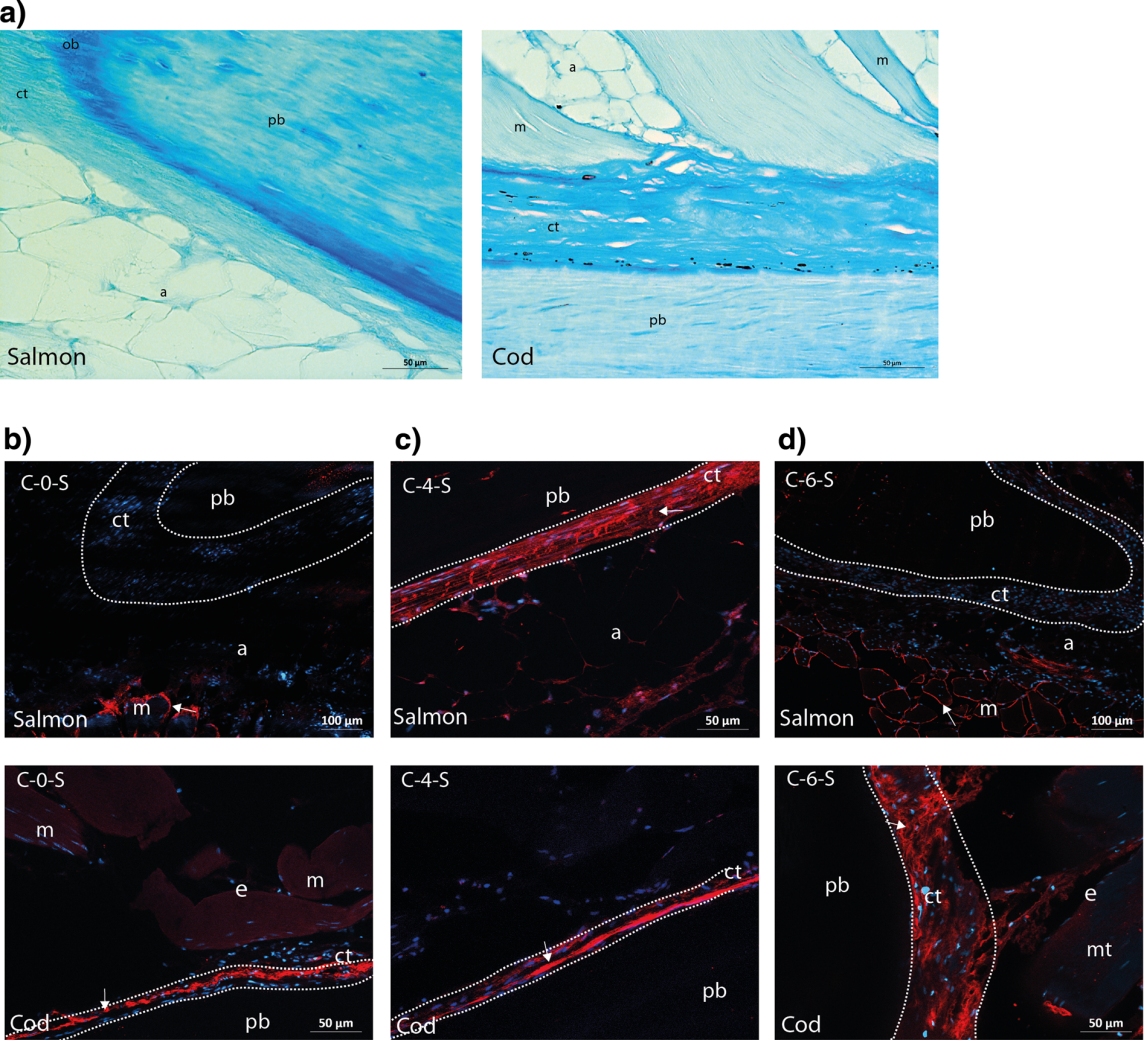
Sulphated components at different positions are present in the attachment areas in salmon and cod (*upper and lower panels*, respectively). **a** Zn-fixed longitude sections of salmon (*left*) and cod (*right*) were stained using Alcian blue with 0.4 mg MgCl2. The connective tissue surrounding the pin bone was rich in sulphated components. *Scale bars* as indicated. *pb* pin bone; *a* adipose tissue; *ct* connective tissue; *m* muscle tissue. **b**–**d** Zn-fixed longitude sections of pin bone attachment sites were stained with mouse anti-C-0-S, anti-C-4-S and C-0-S (*red*) followed by Alexa Fluor 546-conjugated goat anti-mouse before fluorescence microscopy analyses. Nuclei were stained with DAPI (*blue*). Immunostaining (indicated by *arrows*) show strong staining of C-0-S (**b**) and C-6-S (**c**) epitopes in the endomysia in the muscle tissue in salmon, but no staining in the connective tissue in the attachment site around the pin bone. Immunostaining does, however, demonstrate staining of C-4-S epitopes in the endomysia in the muscle tissue as well as staining in the CT in the attachment site around the pin bone (**d**). The immunostaining in cod on the other hand (*lower panels*) show labelling in the CT for all the sulphated epitopes. *pb* pin bone, *a* adipose tissue, *ct* connective tissue, *m* muscle tissue, *e* endomysium. *Dotted areas* denote CT close to the pin bone

In order to identify sulphated components in the CT area close to the pin bone, we stained the pin bone areas with Alcian Blue. This solution, at certain concentrations, stains only negatively charged groups such as the sulphated proteoglycans (Scott and Dorling 1965). Sulphated proteoglycans were present within the pin bone, in the CT as well as in the endomysium and perimysium of the muscle. However, they were most highly stained in the area of CT closest to pin bone in both salmon and cod (Fig. 5a). Furthermore, immunohistochemical analysis showed a different glycosaminoglycan (GAG) epitope distribution and expression in salmon and cod (Figs. 5b–d and S1A–G), summarised in Table 3.

**Table 3:** Expression and distribution of GAG epitopes in muscle tissue, adipose tissue and CT close to the pin bones

GAG epitope	Salmon			Cod		
	Muscle	Adipose	CT	Muscle	Adipose	CT
C-0-S	+	+	−	(+)	n.a.	+
C-4-S	+	+	+	+	n.a.	+
C-6-S	+	+	−	+	n.a.	+

Scored based on expression pattern in Figs. 5b–d and S1A–G

*n.a.* not analysed

**Fig. 6:**
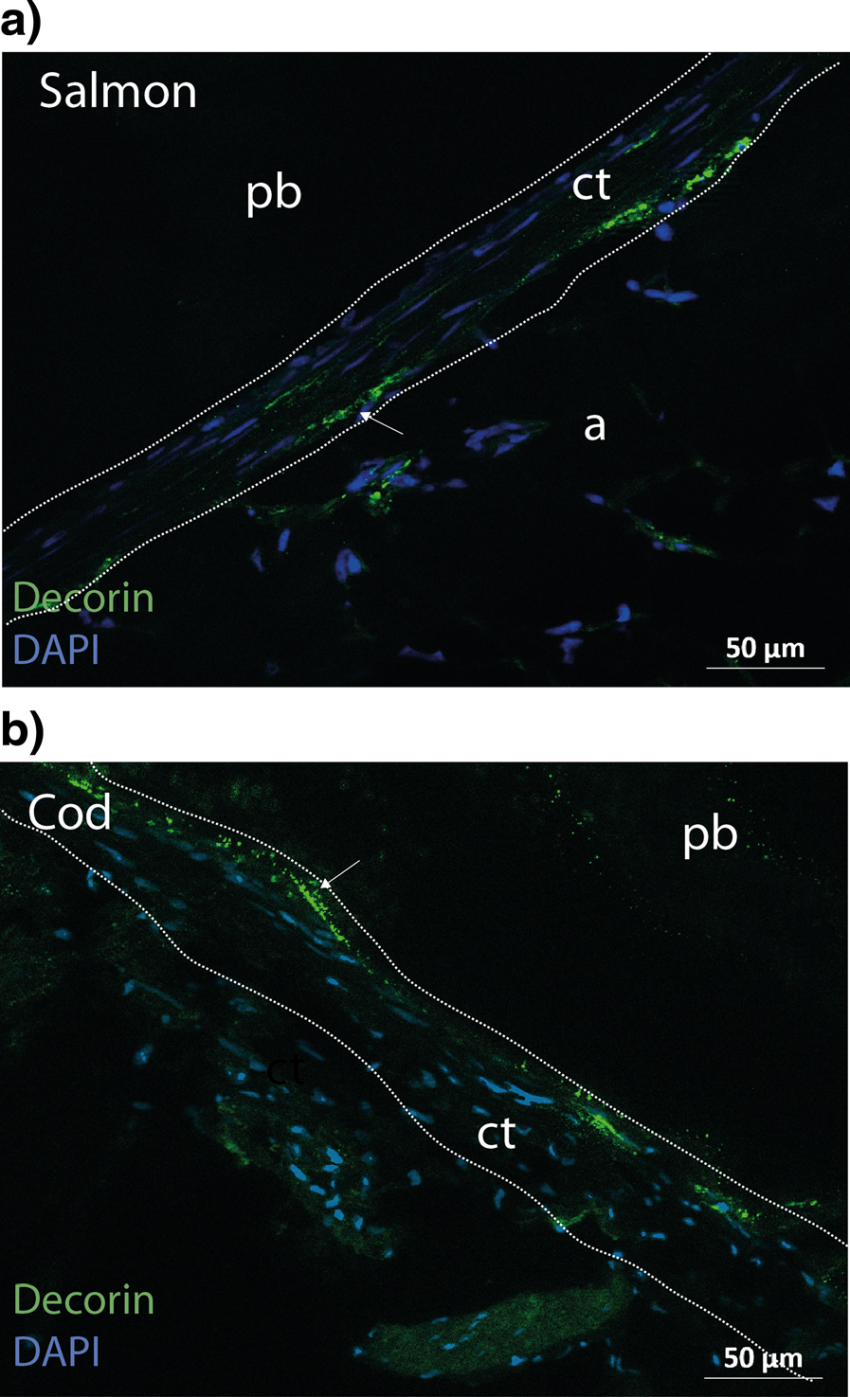
Decorin is present in the CT of salmon and cod. **a**, **b** Zn-fixed longitude sections of pin bone attachment sites in salmon and cod were stained with sheep anti-decorin (*green*) followed by Alexa Fluor 488-conjugated donkey anti-sheep before fluorescence microscopy analyses. Nuclei were stained with DAPI (blue). Immunostaining demonstrates staining in adipose tissue (**a**) and in the CT binding to adipose tissue in the pin bone area. Note that decorin does not seem to be present in the CT closest to the pin bones. Immunostaining demonstrated decorin in the endomysium and within muscle fibres in cod as well as in the CT closest to the pin bone. Indicated by *arrows*. *Scale bars* as indicated. *Dotted areas* denote CT close to the pin bone

The expression of small leucine-rich PGs (SLRPs), decorin and lumican was investigated using immunohistochemical staining. Definite regions of positive decorin staining were observed in the CT area of both salmon and cod (Fig. 6), though at different locations. In cod, decorin was present in the junction between the pin bone and CT, while in salmon it, was observed in the junction between CT and adipose tissue. Decorin was also observed in the adipose tissue of salmon (Fig. S2A) and in the endomysium as well as within the myofibres in cod (Fig. S2B). When staining for lumican, no positive signal was detected in the CT in salmon and cod (data not shown). In cod, on the other hand, a strong staining was detected in the muscle tissue (Fig. S2C).

### Differences in protein abundance and ECM degradation in the pin bone connective tissue from 0 to 5 days postmortem

For both species, we could demonstrate changes in protein expression patterns from 0 to 5 days postmortem with our gel-based proteomics approach. In the salmon samples, we detected at total of 1423 protein spots on the 2-DE gels (Fig. S3A). Of these, 6 spots were found to be significantly altered (*q* value <0.05) during the storage period, while 67 spots showed significant changes according to the less stringent ANOVA procedure (*p* value <0.05). From the preparative gels, we were able to pick out 33 spots for protein identification using in-gel trypsin digestion and MALDI-TOF/TOF mass spectrometry; however, only 6 spots were successfully identified (Table 4). These included proteins potentially involved in stress response (i.e. HSP11 and DJ-1 precursor), aerobic respiration (cytochrome b-c1 complex subunit 1), gluconeogenesis (FBP2), purine and pyrimidine metabolism (thymidine phosphorylase) and protein-protein interaction (Enigma LIM domain protein). For cod, we detected 1262 protein spots on the 2-DE gels (Fig. S3B). Of these, 35 spots had a significant *q* value (*q* < 0.05) indicating changes during the storage period, while 146 spots were significant altered (*p* < 0.05). From the preparative gels, 16 spots were excised for protein identification, but none of these were successfully identified.

**Table 4:** Proteins showing significant changes (*p* < 0.05) in abundance from 0 to 5 days postmortem in the pin bone CT of salmon

Spot no.	Protein (source)	NCBI acc. no.	Matched pep./seq.cov (%)	Ratio 1 h:5 days
2194	Cytochrome b-c1 complex subunit 1, mitochondrial precursor (*Salmo salar*)	gi|224587341	7/11	1.86
2259	Thymidine phosphorylase (*Salmo salar*)	gi|213511662	7/17	0.59
2570	Fructose-1,6-bisphosphatase isozyme 2 (*Salmo salar*)	gi|213510876	10/39	0.46
3163	Heat shock protein Hsp-16.1/Hsp-16.11 (*Salmo salar*)	gi|218931126	6/45	4.81
3167	Enigma LIM domain protein-like (*Salmo salar*)	gi|213515380	9/44	2.25
3500	DJ-1 precursor (*Salmo salar*)	gi|226442872	4/23	0.54

**Fig. 7:**
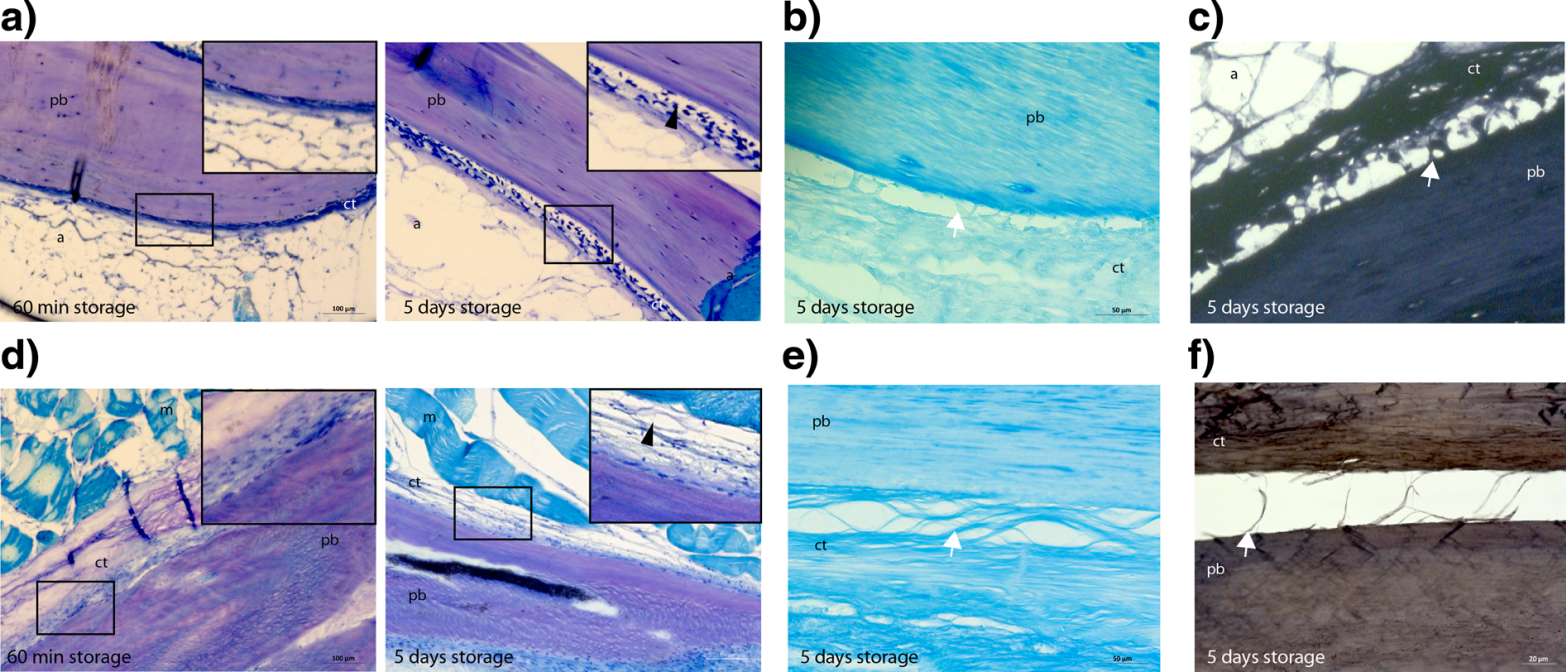
The evolution of CT degradation during storage on ice in salmon (**a**–**c**) and cod (**d**–**f**). **a** Toluidine blue staining of the degradation of the CT in salmon after 1-h storage (*left*) and 5-day storage (*right*). *Arrowheads* in insert (higher magnification of framed area) indicate degradation of CT. **b** Alcian blue with 0.4-mg MgCl_2_ staining show degradation of sulphated components during 5-day storage. **c** Verhoeff’s haematoxylin staining of elastin degradation during 5-day storage. **d**–**f** Staining in cod as described for salmon (**a**–**c**). *Arrows* show globular- versus thread-like degradation. *Scale bars* as indicated. *pb* pin bone, *a* adipose tissue, *ct* connective tissue, *m* muscle tissue

In both salmon and cod, we observed that the CT close to the pin bones began to decay during storage (Fig. 7). The CT was completely dissolved from the pin bones, except for a few attachment points (Fig. 7a, d). The staining of PGs and elastin with Alcian Blue and Verhoeff’s haematoxylin respectively suggested degradation of these structures during loosening of pin bones from the CT in both salmon (Fig. 7b, c) and cod (Fig. 7e, f). Further, the staining also demonstrated a different degradation pattern of the CT, as could be observed as a globular- versus a thread-like structure in salmon and cod respectively.

## Discussion

In the present study, we have demonstrated that the pin bones are attached to muscle and fat in salmon and only to muscle in cod. We also identified various ECM structures that potentially are involved in the firm attachment of pin bones, the CT composition and degradation. The results show that there are major differences between salmon and cod and also that the CT composition enclosing the pin bones differs from the CT profile in the surrounding muscle tissue. Such knowledge is valuable for fish industries when developing methods for automatic removal of bones. Pin bones of salmon and cod have similar structures that are formed in different tissue environments, and this is reflected in their transcriptome. While almost no change in muscle-specific genes in the attachment area of the pin bones compared to the reference muscle sample was observed in salmon, this group was the largest among differentially expressed genes in cod, suggesting rearrangement of muscle structure. Salmon pin bones are submerged in an adipose tissue. This may account for slightly higher expression of genes involved in lipid and steroid metabolism. This may also explain some of the differences observed in pulling force necessary to remove the pin bones in cod and salmon. The transitions between CT and adipose tissue contain weaknesses, and fragmentation often occurs in these transitions.

### The protein composition in the pin bone CT changes during postmortem storage

In our gel-based proteomics approach, we chose to apply a tree-step extraction protocol on the pin bone connective tissue samples. The reasoning behind this was to remove the easily soluble proteins and potentially remaining muscular proteins (that are salt soluble) in order to focus on the CT components. The proteome analysis indicates that many different protein species are present in the pin bone CT of both salmon and cod. The protein spot pattern for the two species differs considerably; however, there are also some similar protein spot patterns. Both species show a relatively large number of protein changes during storage, indicating that the pin bone CT is subjected to multiple postmortem changes. The data from salmon indicate an increase in fructose-1,6-bisphosphatase, a key regulator enzyme of gluconeogenesis, and the production of the start intermediate fructose-6-phosphate and possible reduced mitochondrial activity by reduced amount of cytochrome b-c1 complex. Biochemical changes play an important role for the texture of fish fillets, where acidification postmortem from anaerobic glycolysis resulting in low final pH has been associated with denaturation of proteins, increased proteolysis and reduced CT strength (Torgersen et al. 2014). An association between soft flesh of Atlantic salmon and massive intracellular glycogen accumulation in and between the muscle fibre (the CT) has previously been reported, coinciding with swollen and degenerated mitochondria, myocyte detachment and degradation in connective tissue. The gluconeogenesis pathway is important in the GlcNAc modification of proteins, and whether GlcNAc of proteins and transcription factors is important during connective tissue synthesis (proteoglycans)/enzyme activities would be an interesting aspect in further studies of postmortem processes. Due to the very low protein spot identification success rate of cod in this study, we cannot make any comparison of the specific changes occurring during the postmortem storage period for cod.

### The composition and degradation of the CT enclosing the pin bones during storage

The CT enclosing the pin bones in both cod and salmon is composed of strong structural fibre components such as collagen and elastin, in addition to weaker structural proteins, PGs and lectin-binding proteins. Sialic acid and *N*- acetylglucosaminyl residues are found in lectins, which are carbohydrate-binding proteins that are highly specific for sugar moieties found on the surface of cells. They often bind to soluble extracellular and intracellular glycoproteins. The fact that the CT surrounds the whole pin bone in both salmon and cod can be one of the reasons that early pin bone removal after slaughter is difficult. Elastin is one of the strongest structural components contained in the CT and is made by linking tropoelastin proteins, resulting in insoluble, durable cross-linked complexes. Collagen was also present in the CT surrounding the pin bones. The CT is constantly undergoing changes in response to cellular stimuli, with a well-adjusted interplay between synthesis and deposition of CT components on one hand and their proteolytic breakdown on the other. This is a highly regulated process where proteolytic enzymes, e.g. matrix metallic proteinases (MMPs) and cathepsins are involved. Our array results demonstrated an upregulation of collagens and collagen degrading mmp2 in the CT in the pin bone area, and this suggests active remodelling.

Another major group in the CT is PGs. These are proteins with sugar chains, also called GAG chains, covalently attached to the core proteins and can be divided into different subtypes based on structure and sulphation pattern (Schaefer and Schaefer 2010). There exists four types of covalently attached sulphated GAG chains, dermatan (DS), chondroitin (CS), keratin (KS) and heparan sulphate (HS), in addition to the non-sulphated GAG hyaluronan. PGs enclose the pin bones, and their sulphated sugar groups enable them to bind a variety of proteins and, as such, regulate the structure and turnover of CT. Sugar-protein interactions have been shown to be important for the firmness and attachment of CT in skeletal muscle (Hannesson et al. 2007; Tingbo et al. 2005, 2006, 2012a). The sulphation pattern influences the binding properties of the GAGs and, thus, the overall function of the PGs. Our experiments show that the sulphation pattern is different in salmon and cod (see Fig. 5 and Table 3). While the CT of both species contains CSPGs with C-4-sulphation, PGs with C-0- and C-6 sulphation are absent in the CT of salmon. C-4-S and C-6-S sulphation have opposite effects on cell adhesion, and while C-6-S increases adhesion, C-4-S on the other hand reduces it (Zou et al. 2004). This pattern could be a possible reason for the higher pulling force necessary to remove pin bones in cod compared to salmon (Akse and Tobiassen 2002;. Esaiassen and Sørensen 1996; Westavik 2009). CS are also important in regulating of proteinase activities during matrix remodelling (Georges et al. 2012), where C-4 sulphation (and not C-6-S) has been shown to increase gelatinase A activation (Iida et al. 2007). Gelatinase A, also called MMP2, cleaves type IV collagen, denatured collagen (gelatin) and other ECM components, such as fibronectin, aggrecan, elastin, laminin and collagen I, V, VII and X.

Decorin and lumican are members of a family of SLRPs that contains DS and KS respectively and interacts with fibril forming collagens. Decorin is associated with the formation of thicker and stronger collagen fibrils, whereas lumican is associated with thinner and weaker collagen fibril (Kalamajski and Oldberg 2010). In our study, decorin was present in the CT close to the pin bone in cod and could reflect thicker and more collagenase-resistant collagen fibrils, compared to salmon where decorin rather was present in the junction between adipose tissue and CT. Furthermore, our data demonstrate degradation of CT close to pin bone in salmon, whereas the disruption and degradation of CT in cod occurs further out in the CT, resulting in the thread-like structure observed (compare the degradation of CT in salmon and cod in Fig. 7). Lumican, which is associated with thinner collagen fibrils and a weaker matrix, is not present in the CT around the pin bone in cod. It was, however, detected in connective tissue of skeletal muscle, which is in line with previous data (Tingbo et al. 2012b). When staining for lumican in salmon, no positive signal was detected. The reason for this could be that lumican is not present in neither muscle, adipose nor CT or that the antibody used in this study does not recognize salmon lumican. The SLRPs influence the morphology of the collagen fibrils and the organization of the CT and, thereby, the mechanical properties of the tissue (Kalamajski and Oldberg 2010). Knockout studies in mice have revealed a phenotype with abnormal collagen fibril morphology with fragile skin and tendon, suggesting that decorin stabilizes the fibrillary matrix in vivo, influencing collagen fibril growth and matrix assembly. (Danielson et al. 1997). SLRPs have also been suggested as regulators of intermolecular cross-linking of collagen, thereby determining mechanical properties and degradability of collagen fibrils (Kalamajski and Oldberg 2010). They also appear to limit access of the collagenases to their unique cleavage sites, protecting the collagen fibrils from proteolytic cleavage (Kalamajski and Oldberg 2010).

The collagen organization, type of SLRP present and sulphation modification of the GAG chains differ between salmon and cod, resulting in a CT in cod that are more resistant to enzymatic degradation compared to the CT in salmon. This could be an important information for the industry when developing and optimizing methods for removing pin bones. Disruption in maintenance of collagen fibril placement might be expected to modify shape and destabilize the CT, and any change in sulphation resulting in a decrease of fibril-to-fibril stability and matrix composition might affect CT to a considerable degree and, as such, could this information be important for pin bone removal. Proteolytic enzymes might contribute to loosen the pin bones (Vargova et al. 2012), and identifying such enzymes and their inhibitors would possibly be central in future work of pin bone removal. Enzyme activities are regulated by various factors including pH, temperature and ion strength (Vargova et al. 2012; Georges et al. 2012; Larsen et al. 2008; Esaiassen and Sørensen 1996). Processes that affect these factors could have impact on the loosening, and thus, increase the decay time period (Larsen et al. 2008). The degradation occurs next to the pin bone, and it should be possible to optimize the process both before the slaughter and on the processing line with regard to controlling/accelerating natural degradation around pin bones, and thus make it possible to extract them sooner after slaughter and at the same time avoid injury to muscle fillet.

## Electronic supplementary material

Figure S1 Sulphated components in muscle and adipose tissue in salmon and cod. A-E: Zn-fixed longitude sections of pin bone attachment sites in salmon were stained with mouse anti-C-0-S, anti C-4-S, and C-0-S (red) followed by Alexa 546-conjugated goat anti-mouse before fluorescence microscopy analyses. Immunostaining show strong staining of C-0-S, C-4-S and C-6-S epitopes in the endomysia and the myocommatta in the muscle tissue and in the extracellular matrix around adipose tissue. F-G: Zn-fixed longitude sections of pin bone attachment sites in cod were stained with mouse anti C-4-S, and C-6-S (red) followed by Alexa 546-conjugated goat anti-mouse before fluorescence microscopy analyses. Immunostaining show strong staining of C-6-S epitopes in the endomysia and the myocommatta in the muscle tissue C-4-S show strong staining in the myocommatta in the muscle, but not in the endomysium. *pb* pin bone; *ct* connective tissue; *m* muscle tissue; *mc* myocommata; *e* endomysium;*a* adipose tissue. Indicated by arrows. (GIF 199 kb)

(TIFF 19054 kb)

Figure S2 Extracellular matrix components are present in muscle tissue of salmon and cod. A-D: Zn-fixed longitude sections of pin bone attachment sites in salmon and cod were stained with sheep anti-decorin (green) followed by Alexa 488-conjugated donkey anti-sheep before fluorescence microscopy analyses. A: Immunostaining show staining around fat cells A), and in the connective tissue binding to adipose tissue in the pin bone area. Note that decorin does not seem to be present in the connective tissue closest to the pin bones. B: Immunostaining show decorin in the endomysium and within muscle fibres in cod, as well as in the connective tissue closest to the pin bone. Indicated by arrows. C: Zn-fixed longitude sections of pin bone attachment sites in cod were stained with mouse anti-laminin (green) followed by Alexa 488-conjugated goat anti-mouse before fluorescence microscopy analyses. Laminin is present in muscle fibres and in the connective tissue around the pin bones. D) Zn-fixed longitude sections of pin bone attachment sites in cod were stained with mouse anti-lumican (green) followed by Alexa 488-conjugated goat anti-mouse before fluorescence microscopy analyses. Lumican is not present in the connective tissue around the pin bones. *pb* pin bone; *a* adipose tissue; *ct* connective tissue; *m* muscle tissue. (GIF 219 kb)

High Resolution Image (TIFF 6790 kb)

Figure S3 Representative 2-DE gel images of proteins extracted from salmon and cod pin bone connective tissue (pI 5-8, 12% acrylamide). A: Salmon pin bone connective tissue sample at 0 days post-mortem. B: Cod pin bone connective tissue sample at 0 days post-mortem. Protein spots with significant (q < 0.05) change in abundance during post-mortem storage are indicated and numbered. (GIF 176 kb)

High Resolution Image (TIFF 10089 kb)

Table S1 Genes with differential expression in the pin bone areas in salmon and cod. Samples collected immediately after slaughter were analyzed with oligonucleotide microarrays, bone free muscle was used as reference. Since difference between anterior and posterior samples was minor, results were merged (*n* = 8 in each species). Data are fold (pin bone to bone free muscle ratio). Genes selected by criteria >2-fold and *p* < 0.01 (one sample t-test) are grouped by their functional roles (muscle-specific and extracellular proteins, regulators of differentiation, immune and stress responses, basic cellular processes, metabolic pathways). The numbers of genes from the functional groups are in Table 2, genes with greatest expression differences are shown here. (XLSX 29 kb)

## Abbreviations

ECM: Extracellular matrix
CT: Connective tissue
GAGs: Glycosaminoglycans
PGs: Proteoglycans
