# Supplementary figures and images for: The role of extracellular matrix components in pin bone attachments during storage—a comparison between farmed Atlantic salmon (Salmo salar) and cod (Gadus morhua L.)
Source: Fish Physiol Biochem. 2016 Nov 2;43(2):549–62. doi: 10.1007/s10695-016-0309-0 (PMC5374190; doi:10.1007/s10695-016-0309-0)

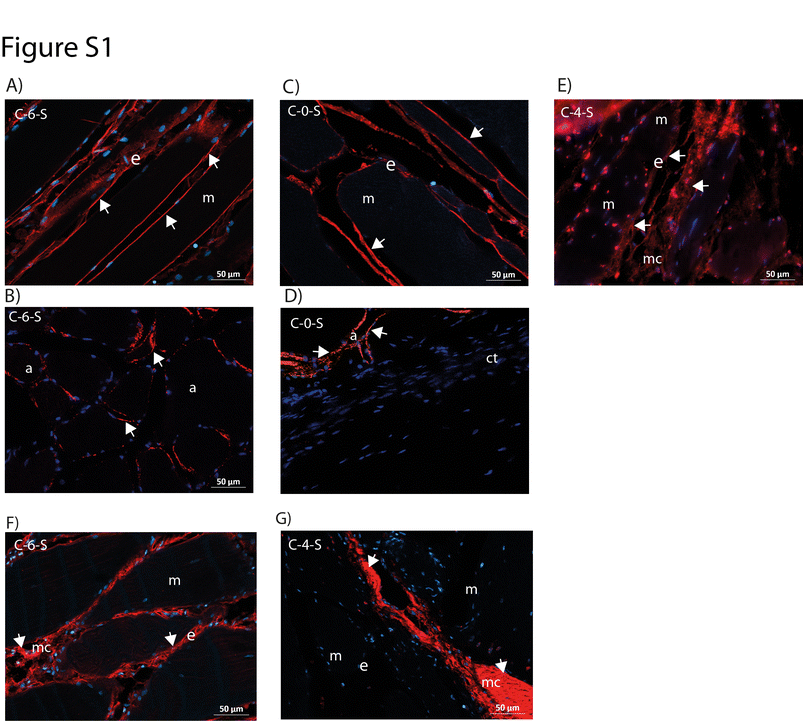

Supplement: Supplementary file 1 — Sulphated components in muscle and adipose tissue in salmon and cod. A-E: Zn-fixed longitude sections of pin bone attachment sites in salmon were stained with mouse anti-C-0-S, anti C-4-S, and C-0-S (red) followed by Alexa 546-conjugated goat anti-mouse before fluorescence microscopy analyses. Immunostaining show strong staining of C-0-S, C-4-S and C-6-S epitopes in the endomysia and the myocommatta in the muscle tissue and in the extracellular matrix around adipose tissue. F-G: Zn-fixed longitude sections of pin bone attachment sites in cod were stained with mouse anti C-4-S, and C-6-S (red) followed by Alexa 546-conjugated goat anti-mouse before fluorescence microscopy analyses. Immunostaining show strong staining of C-6-S epitopes in the endomysia and the myocommatta in the muscle tissue C-4-S show strong staining in the myocommatta in the muscle, but not in the endomysium. pb pin bone; ct connective tissue; m muscle tissue; mc myocommata; e endomysium;a adipose tissue. Indicated by arrows. (GIF 199 kb) [file 10695_2016_309_Fig8_ESM.gif]

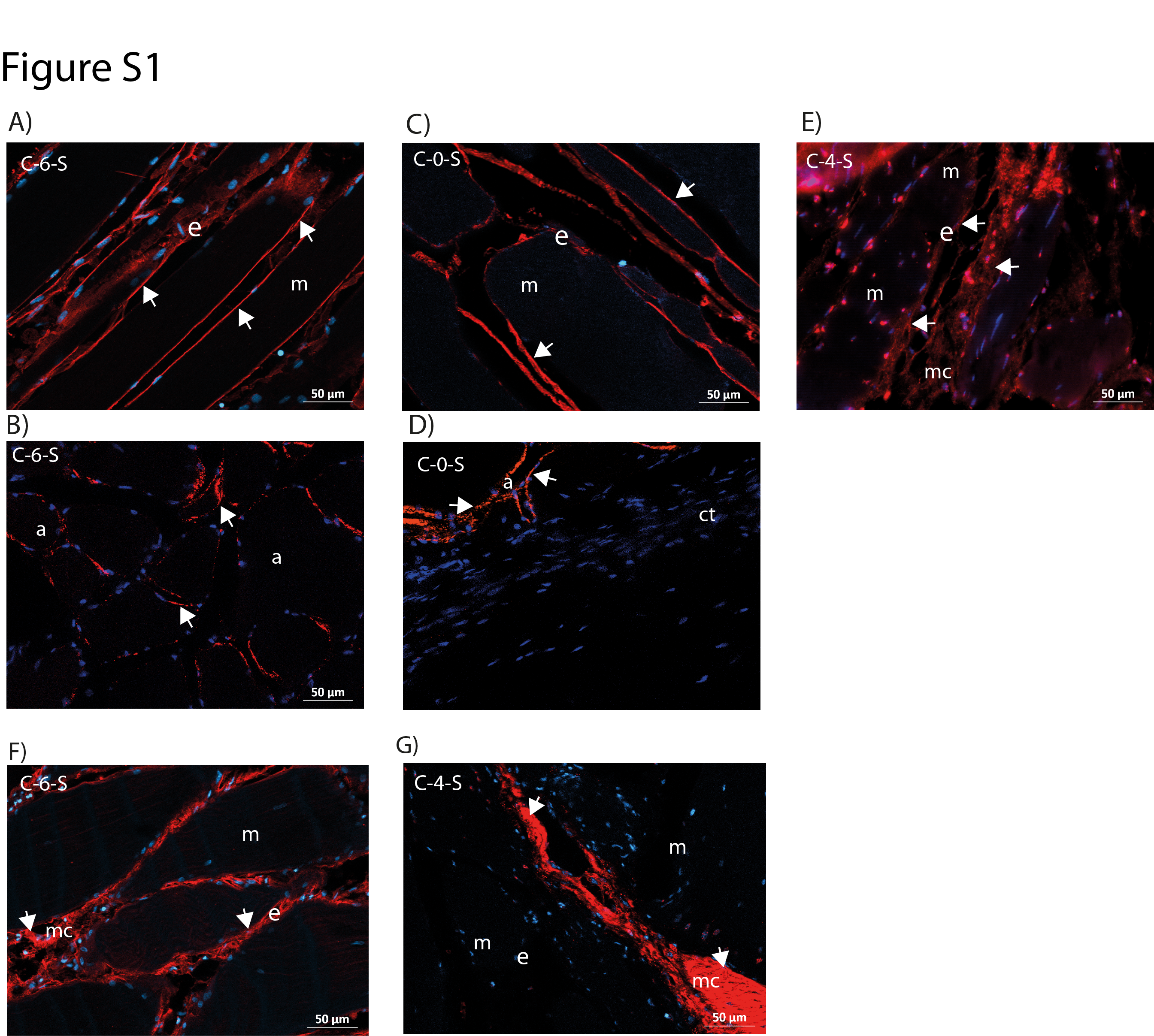

Supplement: Supplementary file 2 — (TIFF 19054 kb) [file 10695_2016_309_MOESM1_ESM.tif]

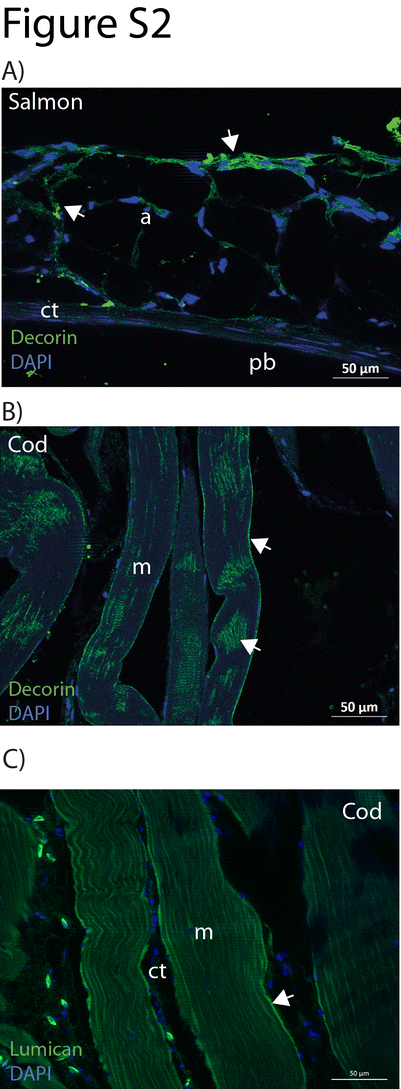

Supplement: Supplementary file 3 — Extracellular matrix components are present in muscle tissue of salmon and cod. A-D: Zn-fixed longitude sections of pin bone attachment sites in salmon and cod were stained with sheep anti-decorin (green) followed by Alexa 488-conjugated donkey anti-sheep before fluorescence microscopy analyses. A: Immunostaining show staining around fat cells A), and in the connective tissue binding to adipose tissue in the pin bone area. Note that decorin does not seem to be present in the connective tissue closest to the pin bones. B: Immunostaining show decorin in the endomysium and within muscle fibres in cod, as well as in the connective tissue closest to the pin bone. Indicated by arrows. C: Zn-fixed longitude sections of pin bone attachment sites in cod were stained with mouse anti-laminin (green) followed by Alexa 488-conjugated goat anti-mouse before fluorescence microscopy analyses. Laminin is present in muscle fibres and in the connective tissue around the pin bones. D) Zn-fixed longitude sections of pin bone attachment sites in cod were stained with mouse anti-lumican (green) followed by Alexa 488-conjugated goat anti-mouse before fluorescence microscopy analyses. Lumican is not present in the connective tissue around the pin bones. pb pin bone; a adipose tissue; ct connective tissue; m muscle tissue. (GIF 219 kb) [file 10695_2016_309_Fig9_ESM.gif]

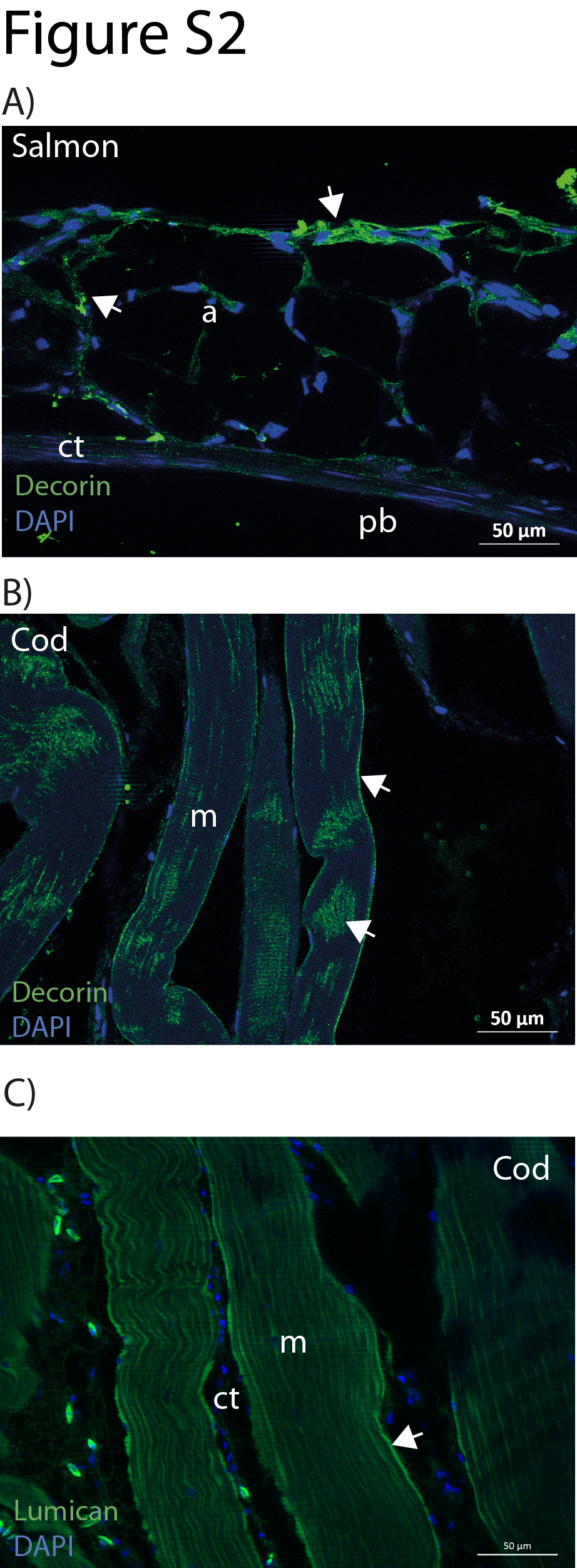

Supplement: Supplementary file 4 — High Resolution Image (TIFF 6790 kb) [file 10695_2016_309_MOESM2_ESM.tif]

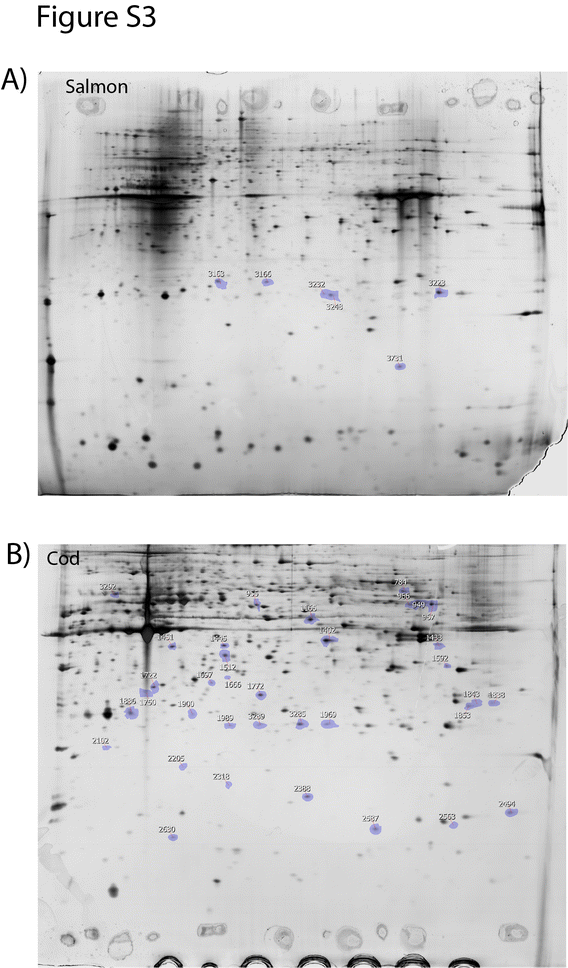

Supplement: Supplementary file 5 — Representative 2-DE gel images of proteins extracted from salmon and cod pin bone connective tissue (pI 5-8, 12% acrylamide). A: Salmon pin bone connective tissue sample at 0 days post-mortem. B: Cod pin bone connective tissue sample at 0 days post-mortem. Protein spots with significant (q < 0.05) change in abundance during post-mortem storage are indicated and numbered. (GIF 176 kb) [file 10695_2016_309_Fig10_ESM.gif]

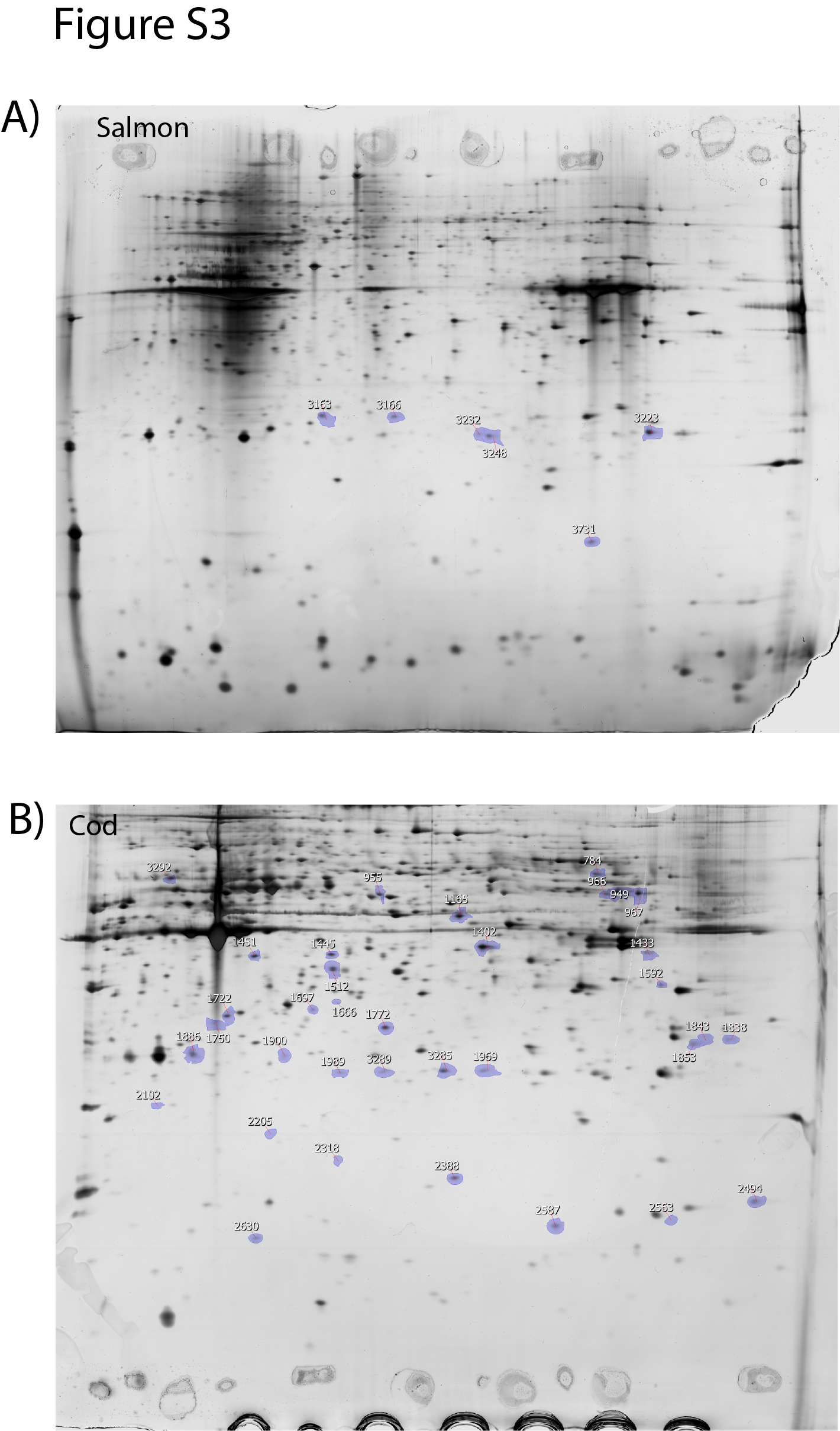

Supplement: Supplementary file 6 — High Resolution Image (TIFF 10089 kb) [file 10695_2016_309_MOESM3_ESM.tif]
